# Quality of life improvements and clinical assessments in kidney transplant recipients undergoing pegloticase treatment for uncontrolled gout: findings of the phase 4 PROTECT clinical trial

**DOI:** 10.3389/fimmu.2025.1516146

**Published:** 2025-03-13

**Authors:** Abdul Abdellatif, Lin Zhao, Katie Obermeyer, Zana Vranic, Brad A. Marder, John D. Scandling

**Affiliations:** ^1^ Department of Medicine, Division of Nephrology at CLS Health and Baylor College of Medicine, Houston, TX, United States; ^2^ Rare Disease Unit, Amgen Inc. (formerly Horizon Therapeutics), Thousand Oaks, CA, United States; ^3^ Division of Nephrology, Stanford School of Medicine, Stanford, CA, United States

**Keywords:** gout, kidney transplant, urate, quality of life, renal function, blood pressure

## Abstract

**Introduction:**

Gout is 12-times more prevalent in kidney transplant (KT) recipients than in non-transplanted population. We report quality-of-life (QOL) and clinical assessment findings from the PROTECT trial examining pegloticase efficacy and safety in KT recipients with uncontrolled gout.

**Methods:**

Patients with serum urate (SU) ≥7 mg/dL, oral urate-lowering therapy refractory/intolerant, and with one of the following were enrolled: ≥2 flares/year, unresolving tophi, or chronic gouty arthritis. Patients were ≥1 year post-transplant, with a graft eGFR ≥15 ml/min/1.73m^2^ and received stable immunosuppression. Pegloticase was administered for 24 weeks. QOL endpoints included the Health Assessment Questionnaire (HAQ; Disability Index [DI], Health, Pain) and Physician Global Assessment (PhGA) of Gout. Key clinical assessments included proportion of patients with resolution of ≥1 tophus and change from baseline in blood pressure (BP) at Week 24.

**Results:**

Twenty KT recipients (85.0% male, age: 53.9±10.9 years, BMI: 30.6±7.2 kg/m^2^, eGFR: 45.8±11.9 ml/min/1.73 m^2^, time since kidney transplant: 14.6±6.9 years) were included. The primary endpoint was achieved with 89% of patients reaching and maintaining a SU of <6 mg/dL during Month 6. Meaningful improvements occurred over 24 weeks of treatment in all QOL measures (mean [95% CI] change from baseline: HAQ-DI: -0.3 [-0.6, 0.1], HAQ-Pain: -35.5 [-54.5, -16.5], HAQ-Health: -22.4 [-39.5, -5.2], PhGA: -2.4 [-3.7, -1.1]) and clinical assessments (≥1 tophus resolved: 3 of 7 with tophi at baseline [42.9%]; change from baseline in mean arterial BP: -6.8 [-12.5, -1.0] mmHg).

**Conclusions:**

Given the high prevalence of uncontrolled gout in KT recipients, proper SU management is of particular importance. Additionally, intensive urate-lowering with pegloticase may have clinical and QOL benefits.

## Introduction

The kidney is the most common solid organ transplanted in the United States (1, 2) and the treatment of choice for patients with end-stage renal disease (3). Because urate is primarily eliminated from the body via renal excretion, patients with compromised renal function are at an increased risk for both hyperuricemia and gout (4). Calcineurin inhibitors (CNIs) including cyclosporin and tacrolimus are the most commonly used immunosuppressants after kidney transplantation and are known to decrease urinary clearance of uric acid, leading to hyperuricemia that can contribute to the development of gout (5). Approximately 1 in 8 kidney transplant (KT) recipients (13%) develop gout, which is 12-times greater than the gout prevalence in the general non-transplanted US population (2, 6). Uncontrolled gout can have systemic consequences and a negative impact on the patient quality of life (7–9). Gout is also an independent risk factor for CKD progression (10) and end-stage renal disease (ESRD) (11), with higher SU levels leading to an increased risk (12, 13). In KT recipients specifically, gout is associated with worse quality-of-life (QOL) (14, 15) and increased renal graft failure rate in KT recipients (16, 17).

Pegloticase is a recombinant PEGylated uricase that effectively lowers SU in gout patients with oral ULT inefficacy, intolerance, or contraindication and is currently only available by intravenous route. Pegloticase catalyzes the conversion of urate in the serum to allantoin, an inert and highly water-soluble molecule that is readily excreted by the kidneys. Immunosuppressive therapy has been a fundamental component of organ transplantation to prevent graft rejection and to improve graft survival. Although patients with organ transplant were excluded from Phase 3 studies of pegloticase (monotherapy), nearly half the patients had stage 3 and 4 chronic kidney disease (CKD) but showed safety and efficacy comparable to the general study population (18). These findings suggested the potential of pegloticase to rapidly deplete monosodium urate deposits, even when urinary urate excretion is impaired.

However, the immunogenicity rate was high in Phase 3 trials, with antibodies to the polyethylene glycol moieties (anti-PEG) antibodies detected in 89% of patients, which can limit the safety and efficacy of pegloticase (18). The larger MIRROR randomized controlled trial (MIRROR RCT) demonstrated that methotrexate (MTX), when administered as co-therapy to pegloticase, increased treatment sUA-lowering response rate (71%) compared with placebo (39% during Month 6), decreased IR risk (4% vs. 31% through Month 6), and decreased *de novo* anti-drug antibody (ADA) formation (23% vs. 50% through Month 6), with substantially lower titers in the MTX group (19). Moreover, a recent literature review reported that increased pegloticase response rates (83% aggregated response) were observed when pegloticase was co-administered with immunomodulators including methotrexate (87.5%), MMF (86%), leflunomide (67%), and azathioprine (64%) (20). Based on the encouraging results of the MIRROR, methotrexate is now recommended as co-therapy to pegloticase based on higher rates of urate-lowering efficacy and lowered risk of infusion reaction compared to pegloticase monotherapy.

The Phase 4, multisite, open label PROTECT trial was specifically designed to evaluate the safety and efficacy of pegloticase in KT patients on immunosuppression with chronic gout refractory to conventional urate lowering therapy. The primary findings of the study have been previously published and demonstrated a high rate of urate-lowering efficacy with pegloticase in KT recipients with no new safety signals (21). In this trial, all patients were on a stable immunosuppression regimen to prevent graft rejection.

Data on QOL in patients with uncontrolled gout are limited. However, studies have shown an association between frequency of acute gout flares, presence of tophi, work productivity, disability, pain, and an impact on QOL (14, 15). Given that gout patients who maintain serum urate levels (SU) <6 mg/dL have fewer flares and can experience resolution of tophi (22, 23), SU management in KT recipients with gout could lead to QOL improvements. Because the use of oral urate-lowering therapies (ULTs) can be limited by renal function and drug interactions in KT recipients, other SU-lowering treatments are important for the KT population. Here, we further report the secondary endpoints of the PROTECT trial, which focus on changes in renal function and patient QOL in KT patients during and after 6-months of pegloticase treatment.

## Patients and methods

The Phase 4, multisite, open label PROTECT clinical trial (NCT04087720) was conducted in accordance with the principles of the Declaration of Helsinki. A local institutional review board or ethics committee at each study site approved the protocol. Written informed consent was obtained from all patients to participate in this study prior to performing any study examinations or procedures. Study methods have been previously described in full (21), but are described here in brief for completeness.

### Patients

This clinical trial included adult KT recipients with uncontrolled gout, defined as SU ≥7 mg/dL, oral ULT inefficacy or intolerability, and satisfying at least 1 of the following gout symptom: evidence of gouty tophaceous deposits (visible unresolved tophi); or recurrent gout flares defined as 2 or more flares in the past 12 months prior to screening, or presence of chronic gouty arthritis. All patients were ≥1-year post-transplant and on a stable immunosuppression regimen for ≥3 months prior to study screening. Patients were required to have a functional graft with an eGFR ≥15 ml/min/1.73 m^2^ and to tolerate low-dose prednisone (<10 mg/day) as gout flare prophylaxis (initiated ≥1 week prior to first pegloticase infusion). Key exclusion criteria included patients with an unresolved severe infection less than 2 weeks prior to Day 1, chronic or active hepatitis B infection, history of hepatitis C virus RNA positivity unless treated and undetectable, history of HIV positivity, G6PD deficiency, congestive heart failure, uncontrolled arrythmia, or uncontrolled hypertension (>160/100 mmHg) at the end of the Screening Period.

### Study design

The PROTECT trial examined the efficacy and safety of pegloticase in adult KT recipients with uncontrolled gout. Enrolled patients who continued to meet all eligibility criteria through the Screening period (≤35 days) entered the 24-week pegloticase treatment period (8 mg infusion every 2 weeks; 12 infusions). All patients received standard flare prophylaxis for ≥1 week prior to first treatment and infusion reaction prophylaxis prior to each pegloticase dose. Patients completed a safety visit via phone or email 30 days after the last pegloticase infusion and a full clinical assessment 3 months after the last pegloticase infusion. Patients were allowed to initiate oral urate-lowering agents after pegloticase discontinuation.

The study’s primary endpoint was the SU-lowering response rate during month 6, defined as SU <6 mg/dL for ≥80% of Weeks 20-24. Key secondary endpoints included mean change from baseline at Week 24 in Health Assessment Questionnaire (HAQ)-Pain and Disability Index (DI). HAQ-Health was also examined but was an exploratory endpoint. Other key exploratory endpoints included the proportion of patients with complete resolution of ≥1 tophus and the change from baseline at Week 24 in Physician Global Assessment (PhGA), eGFR, urine albumin creatinine ratio (UACR), and blood pressure (BP). BP was measured three times (≥2 minutes apart) or more prior to infusion to get readings that differ by < 8 mmHg for SBP and < 5 mmHg for DBP. The mean of these BP measurements was used in data analysis. An independent central reader interpreted study photographs of tophi. For measurable tophi, resolution was defined as a 100% decrease in tophus area, defined as the product of length of the longest dimension and the length perpendicular to the longest dimension. For unmeasurable tophi, resolution was defined as the disappearance of the tophus.

### Procedures

Baseline measurements were collected prior to first pegloticase infusion (Day 1). Study visits occurred every 2 weeks through Week 24. Prior to each infusion, SU, vital signs, and adverse event (AE) information were obtained. HAQ and PhGA measures were also collected at baseline, Weeks 6, 14, 20, and 24 (or End-of-Treatment), and the 3-month follow-up visit. Blood and urine samples were collected for laboratory analyses at baseline, Weeks 2, 4, 6, 8, 10, 14, 22, and 24 (or End-of-Treatment), and the 3-month follow-up visit. Additional non-infusion visits occurred at Weeks 21 and 23 for SU measurement.

HAQ-Pain and HAQ-Health have a scoring range from 0 (no pain/very well health) to 100 (severe pain/very poor health). Both measures have a minimum clinically important difference (MCID) of 10 (24). HAQ-DI has a scoring range from 0 (no disability) to 3 (maximum disability; MCID = -0.22 (25)) and PhGA has a scoring range from 0 (excellent) to 10 (very poor; MCID = 1 (26)). A decrease in these measures represents improvement in the respective areas. UACR levels were classified into three categories to assign an albuminuria grade (A1: <30 mg/g, A2: 30–299 mg/g, A3: ≥300 mg/g) based on measured values (27).

### Statistical methods

#### Sample size and power consideration

A sample size of 20 patients was planned for this study. The primary efficacy endpoint reported previously (21), which was the proportion of patients achieving and maintaining sUA <6 mg/dL for at least 80% of the time during Month 6, was demonstrated to be statistically greater than 43.5% (proportion of responders during Month 6 in Phase 3 pegloticase monotherapy studies as a reference), if at least 14 of 20 (70%) responders were observed. In that case, the lower bound of a 95% confidence interval for the proportion of responders will be about 46%.

#### Study endpoints

Detailed statistical methods for the study, the primary endpoint, safety, pharmacokinetics, and pegloticase immunogenicity are fully described in the primary manuscript (21). Briefly, a sample size of 20 patients was planned for this open-label study (28). Efficacy analyses were performed on the intent-to-treat (ITT) population, defined as all patients who received ≥1 infusion of pegloticase. For the primary endpoint analysis, patients who discontinued treatment prior to Month 6 for COVID-19─related reasons were excluded from analysis.

For secondary (HAQ-Pain, HAQ-DI) and exploratory endpoints (HAQ-Health, tophus size, eGFR, UACR, PhGA, and BP), the change from baseline to each visit was summarized. Continuous variables were summarized using descriptive statistics (number of patients, mean, standard deviation, and 95% confidence interval). Summaries of the mean change from baseline to visits through Week 24 for QOL and renal assessments only results from patients who remained on treatment at that visit were included, to obtain data from patients actively on therapy and exclude patients who discontinued treatment. The 3-month follow-up visit included all subjects who had available data. No adjustments were made for multiplicity in the open-label study. Categorical variables were summarized using frequencies and percentages. Unless otherwise specified, baseline was defined as the last available observation prior to the first dose of pegloticase.

## Results

### Patient baseline characteristics and disposition

The first patient was screened on September 17, 2019 (study start date), and the last patient completed the last visit on September 7, 2021 (study completion date). A total of 20 patients were enrolled and received ≥1 dose of pegloticase (ITT). Of these, 14 patients completed treatment through Week 24. Six patients discontinued pegloticase treatment before completing the 24-week treatment period. The reasons for treatment discontinuation were: sUA stopping rule met in 2 patients indicating lack of SU-lowering efficacy (2 consecutive pre-infusion SU >6 mg/dL after Week 2); voluntary withdrawal by one patient after experiencing an SAE at Week 6 (atrial fibrillation, deemed unrelated to pegloticase by the investigator), and COVID concerns in 3 patients. Overall patient disposition and details on the 18 patients included in efficacy analysis are given in **Supplementary Figure 1**.

Among the 20 enrolled patients, the mean ± SD age was 53.9 ± 10.9 years; 85% were male, and body mass index (BMI) was 30.6 ± 7.2 kg/m^2^. Patients had a 7.9 ± 11.6-year history of gout (time from diagnosis), SU prior to treatment was 9.4 ± 1.5 mg/dL, 55% (11 of 20) had a history of visible tophi, and 90% (18 of 20) had experienced ≥1 gout flare within the 12 months prior to Screening. Mean time from KT averaged 14.6 ± 6.9 years, and mean pre-treatment eGFR was 45.8 ± 11.9 ml/min/1.73 m^2^ (90% [18 of 20] had eGFR <60 ml/min/1.73 m^2^) (**Table 1**).

**Table 1:** Participant demographics and clinical characteristics at baseline.

	Study Population (N=20)
Patient Characteristics	
Age, mean ± SD, years	53.9 ± 10.9
Male sex, n (%)	17 (85)
Race, n (%)	
White	9 (45)
Asian	2 (10)
Black or African American	7 (35)
Other	2 (10)
Body mass index, mean ± SD, kg/m^2^	30.6 ± 7.2
Gout characteristics	
Time since first gout diagnosis, mean ± SD, years	7.9 ± 11.6
Tophi, n (%)	11 (55)
Number of flares per participant in the past 12 months, n (%)	
None	2 (10)
1	1 (5)
≥2-3 ≥4-9	2 (10) 9 (45)
≥10 or more	6 (30)
Serum urate, mg/dL, mean ± SD	9.4 ± 1.5
Immunosuppression regimens*, n (%)	
Tacrolimus/mycophenolate/prednisone	9 (45)
Tacrolimus/prednisone	3 (15)
Cyclosporine/mycophenolate/prednisone	2 (10)
Cyclosporine/azathioprine/prednisone	2 (10)
Mycophenolate/prednisone	2 (10)
Sirolimus/mycophenolate/prednisone	1 (5)
Cyclosporine/prednisone	1 (5)
Baseline kidney transplant characteristics	
Time since kidney transplant, mean ± SD, years	14.6 ± 6.9
eGFR**^†^**, mL/min/1.73m^2^, mean ± SD	45.8 ± 11.9
Chronic kidney disease stage, n (%)	
Stage 2, n (%)	2 (10)
Stage 3a, n (%)	6 (30)
Stage 3b, n (%)	11 (55)
Stage 4, n (%)	1 (5)

*Stable dose ≥3 months pre-trial and continuing during trial.

^†^Last measurement prior to first pegloticase infusion. SD, standard deviation; eGFR, estimated glomerular filtration rate.

All patients were on a stable immunosuppression regimen, with 70% (14 of 20) on triple immunosuppression consisting of a calcineurin inhibitor (CNI) or mTOR inhibitor-based protocol (tacrolimus, cyclosporin, or rapamycin), an antimetabolite (mycophenolate or azathioprine), and prednisone. Overall, 25% (5 of 20) received cyclosporine as part of their regimen (**Table 1**), which was expected considering that CNIs including cyclosporine and tacrolimus are cornerstone immunosuppression agents for kidney transplantation and well known to cause hyperuricemia by reducing renal urate excretion. Other relevant concomitant medications taken prior to, within the pegloticase treatment period, and during follow-up are summarized in **Supplementary Table 1**.

### Primary outcomes and gout-related findings

The primary outcomes of the PROTECT trial have been previously published (21). To summarize, marked and sustained reductions in sUA during ongoing treatment were achieved in patients who completed treatment and also in those who discontinued treatment for reasons other than the SU discontinuation criteria (2 consecutive pre-infusion SU >6mg/dL after Week 2) but completed study follow-up. Overall, 89% (16 of 18 [95% CI: 65.3, 98.6]) of the patients achieved the primary endpoint of maintaining SU <6 mg/dL for ≥80% of the time during Month 6 (Weeks 20-24). Two patients who discontinued treatment prior to Month 6 for COVID-19 reasons were excluded from analysis.

The sUA reduction was observed by the Week 2 visit for responders. The two patients who showed two consecutive sUA values >6 mg/dL were deemed to be non-responders and discontinued treatment per the study protocol. They were among the four patients who did not receive an antimetabolite (mycophenolate or azathioprine) as part of their immunosuppression regimen.

Of the 7 patients with tophi at baseline, 3 (42.9%) had complete resolution of ≥1 tophus at Week 24, 2 (28.6%) had partial resolution of ≥1 tophus at Week 24, and 2 were missing tophi evaluation at Week 24. Only 3 patients had measurable tophi assessed at Week 24, but tophi size (sum of the long axis diameter among all tophi measured) decreased in all 3 patients with mean change from baseline of -43.3 (min=-72.2, max=-10.2) mm. Tophi size continued to decrease through the 3-month follow-up with mean change of -73.4 (min=-127.3, max=-10.2) mm.

### Pegloticase immunogenicity

Detailed immunogenicity findings of the PROTECT paper have been published along with the primary outcomes (21). Briefly, the incidence of anti-PEG antibodies was measured to assess the immunogenicity of pegloticase in the 20 KT recipients. A substantial increase in anti-PEG titers and corresponding decrease in serum pegloticase concentrations was observed only in the two non-responders; notably, all responders had a lower anti-PEG titer and higher serum pegloticase levels. The increase in anti-PEG titer for the two non-responders corresponded with loss of pegloticase exposure and sUA increase. Positive anti-uricase IgG antibodies were only detected in one patient post treatment with a very low titer (<10).

### Renal findings

**Figure 1:**
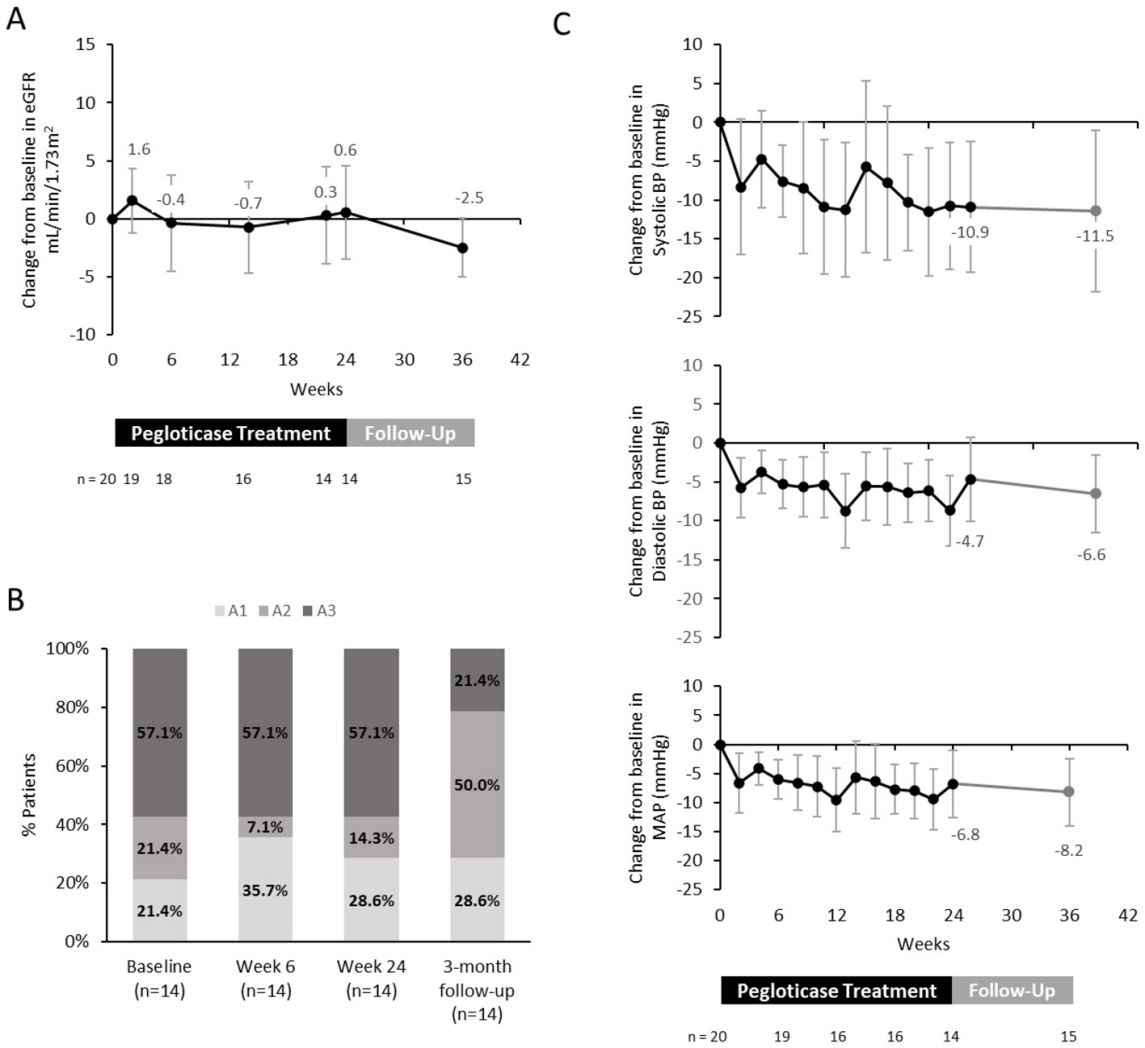
**(A)** Mean change from baseline in eGFR. **(B)** Albuminuria stage in patients who completed pegloticase treatment through Week 24. Only patients with paired baseline/Week 24 measurements were included (A1: <30 mg/g, A2: 30–299 mg/g, A3: ≥300 mg/g (27). **(C)** Mean change from baseline in blood pressure. MAP, mean arterial pressure. Error bars represent 95% confidence interval. All figure parts show available data from patients on treatment.

Renal function was monitored during pegloticase treatment through eGFR, UACR, and BP assessments. At Week 24, eGFR remained stable with a mean (95% CI) change from baseline of +0.6 (-3.5, 4.6) mL/min/1.73 m^2^ (**Table 2**) and continued to remain stable during the 3-month post-pegloticase follow-up period (mean change from baseline: -2.5 (-5.0, 0.1) mL/min/1.73 m^2^; **Figure 1A**). Mean UACR also remained stable, with high variability among the data. Applying the clinical definition of albuminuria classifications (27), albuminuria grade was stable through Week 24 of treatment. However, 5 of the 8 patients (62.5%) with severe albuminuria (A3) at baseline and week 24 showed improvement to moderate albuminuria (A2) at the 3-month follow-up visit (**Figure 1B**).

**Table 2:** Renal parameters and quality of life measures in kidney transplant recipients receiving 24 weeks of pegloticase treatment.

	Baseline Mean ± SD	Week 24 Mean ± SD	Change from Baseline Mean (95% CI)
Renal Function (n=14)			
eGFR, mL/min/1.73m2	43.4 ± 11.4	44.0 ± 10.5	+0.6 (-3.5, 4.6)
UACR, mg/g	893.4 ± 1226.8	876.6 ± 1538.9	-16.7 (-460.8, 427.4)
Blood Pressure (n=14)			
Systolic, mmHg	142.6 ± 15.4	131.7 ± 11.7	-10.9 (-19.3, -2.5)
Diastolic, mmHg	84.8 ± 9.4	80.1 ± 8.9	-4.7 (-10.1, 0.8)
Mean Arterial, mmHg	104.1 ± 10.2	97.3 ± 8.6	-6.8 (-12.5, -1.0)
HAQ (n=13*)			
Disability Index (MCID = -0.22)	1.0 ± 1.0	0.7 ± 0.8	-0.3 (-0.6, 0.1)
Pain Score (MCID = -10)	42.7 ± 29.6	7.2 ± 21.1	-35.5 (-54.5, -16.5)
Health Score (MCID = -10)	39.8 ± 28.7	17.4 ± 29.1	-22.4 (-39.5, -5.2)
PhGA (n=14) (MCID = -1)	5.1 ± 1.5	2.7 ± 2.6	-2.4 (-3.7, -1.1)

*n=14 for HAQ disability index. eGFR, estimated glomerular filtration rate; UACR, urine albumin-to-creatinine ratio; CI, confidence interval;HAQ, Health Assessment Questionnaire; PhGA, Physician Global Assessment of Gout; MCID, minimal clinically important difference; SD, standard deviation. Baseline is presented for patients with on-treatment results available at Week 24.

The mean (95% CI) change from baseline in SBP, DBP, and MAP at Week 24 was -10.9 (-19.3, -2.5), -4.7 (-10.1, 0.8), and -6.8 (-12.5, -1.0) mmHg, respectively (**Table 2**). These BP changes persisted for at least 3-months following pegloticase treatment (**Figure 1C**).

### Quality of life findings

**Figure 2:**
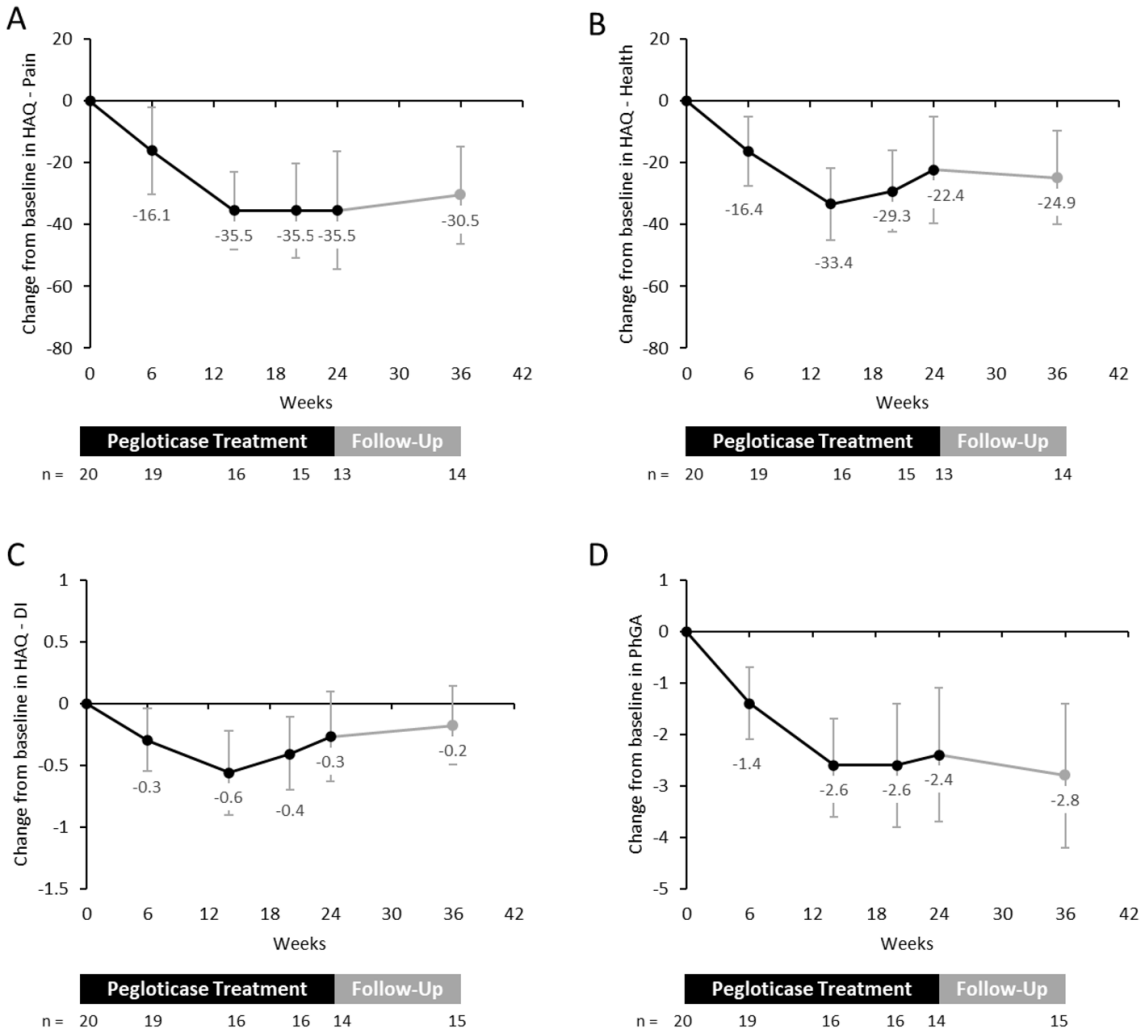
Mean change from baseline in HAQ measures **(A-C)** and PhGA **(D)** in kidney transplant recipients receiving up to 24 weeks of pegloticase therapy. Because HAQ and PhGA scores quantify how much gout impacts a patient, decreases in these measures represent patient improvements. Data is also shown for the 3-month follow-up visit. Error bars represent 95% confidence interval. All figure parts show available data from patients on treatment. HAQ, Health Assessment Questionnaire; DI, Disability Index; PhGA, Physician Global Assessment of Gout.

In patients who received treatment through Week 24, mean (± SD) baseline HAQ-Pain, -DI, and -Health scores were 42.7 (± 29.6), 1.0 (± 1.0), and 39.8 (± 28.7), respectively, indicating high impact of gout on QOL (**Table 2**). All HAQ measures meaningfully improved (decreased) for those who remained on treatment through Week 24 in the pegloticase treatment period. At Week 24, mean (95% CI) change from baseline in HAQ-Pain was -35.5 (-54.5, -16.5) (8 of 13 [61.5%] met MCID); mean change from baseline in HAQ-DI was -0.3 (-0.6, 0.1) (6 of 14 [42.9%] met MCID), and mean change from baseline in HAQ-Health was -22.4 (-39.5, -5.2) (7 of 13 [53.8%] met MCID; **Figures 2A–C**). HAQ-Pain and HAQ-Health improvements persisted through the 3-month follow-up period (**Figures 2A, B**). Mean PhGA at baseline was 5.1 (± 1.5), also indicating a high gout related QOL impact of gout on this population (**Table 2**). At Week 24, the mean (95% CI) change from baseline in PhGA was -2.4 (-3.7, -1.1) (11 of 14 [78.6%] met MCID). As with HAQ measures, the PhGA improvements persisted though the 3-month follow-up period (**Figure 2D**).

## Discussion

Gout and hyperuricemia are highly prevalent in KT recipients due to compromised renal function and commonly used medications, including calcineurin inhibitors (29) and diuretics (30). Further, KT recipients with gout are at an increased risk for graft failure, as indicated by a higher rate of returning to dialysis (17). Unfortunately, managing gout in KT recipients can be challenging, with oral ULT use limited by both renal function and comorbidities (29). Per pegloticase indication, the PROTECT clinical trial enrolled KT patients with chronic gout who have failed to normalize sUA and whose signs and symptoms are inadequately controlled with xanthine oxidase inhibitors at the maximum medically appropriate dose or for whom these drugs are contraindicated.

As published previously, the PROTECT results demonstrated a urate-lowering efficacy rate of 89% during Month 6 of pegloticase treatment in KT recipients with no safety signals specific to KT recipients (21). In previous trials with non-transplant patients, the development of anti-PEG antibodies, but not anti-uricase antibodies, has been linked to infusion reactions, low serum pegloticase concentrations, and loss of urate-lowering response (18, 19). In line with prior studies, non-response to pegloticase in the current PROTECT trial was associated with increased anti-PEG antibody titers and decreased serum pegloticase concentrations. Further, anti-uricase antibodies did not influence pegloticase response rates (21).

The key secondary and exploratory findings of the PROTECT trial presented here provide further insight into potential benefits of pegloticase-induced urate-lowering in this population, showing renal function stability, meaningful QOL improvements, and BP and albuminuria benefits in some patients. Improvements in long-term patient and graft survival remain critical unmet needs for kidney transplant patients. Treatments targeting hypertension and albuminuria could play an important role in this effort by preventing chronic allograft nephropathy and cardiovascular disease (31–33). Notably, systematic reviews and meta-analyses have suggested a favorable effect of urate-lowering therapy on blood pressure (34), establishing the association between high urate levels and high blood pressure. Additionally, hyperuricemia or gout is indicated to have a causal effect on hypertension (35). Both CKD (36) and hypertension (37) are associated with an increased risk of cardiovascular events. Although not completely elucidated, urate-lowering therapy may decrease blood pressure by blocking the renin–angiotensin system (RAS), which is activated in hyperuricemia (38). Previous studies have established a close association between plasma aldosterone levels, which is the end-product of RAS, and the occurrence of hyperuricemia/gout (39). In the current study, SBP, DBP, and MAP slightly decreased during therapy, with notable changes observed 2 weeks after treatment initiation. These decreases were sustained during treatment and through the 3-month post-treatment follow-up visit. These findings are consistent with those during pegloticase use in a non-transplant population with uncontrolled gout (40).

The current study demonstrated renal function stability, as assessed with eGFR and UACR/albuminuria stage evaluation, during pegloticase treatment. In the 3 months following pegloticase treatment, mean eGFR continued to remain stable, but albuminuria showed improvement in 62.5% of patients with severe albuminuria (A3) at baseline (remaining patients had albuminuria stage stability). However, it is unknown if this potential improvement was due to pegloticase treatment, urate-lowering, or other factors.

The high impact of gout on lowering patient QOL is well established, and these effects are more pronounced in both KT recipients (14) and non-transplant patients (18, 26) with uncontrolled gout. Flare frequency (14), tophi presence (14), and gout-related pain (41) heavily contributed to QOL impact, leading to significant loss in work productivity, social activity, and self-care abilities (42). As in non-transplant populations (18, 26), the current study demonstrated QOL gains during pegloticase treatment in KT recipients, with clinically meaningful improvements in HAQ-Health, HAQ-Pain, and HAQ-DI scores during treatment. These improvements largely persisted for at least 3-months following pegloticase treatment. Given the marked reduction in urate burden in some of these patients, as measured by dual-energy computed tomography (43), maintaining SU <6 mg/dL following pegloticase treatment would keep gout flare frequency low based on evidence from prior studies (44, 45). Further, maintaining SU <6 mg/dL would theoretically prevent urate deposits from reforming (urate solubility limit in the serum = 6.8 mg/dL). This would hopefully allow QOL improvements gained during pegloticase treatment to persist over the long-term.

This study was limited by its small sample size, lack of placebo control, open-label design, and heterogeneity of immunosuppression regimens among patients. Uncontrolled gout is a rare form of gout associated with pain and poor quality of life due to due a high burden of monosodium urate accumulation not amenable to oral ULT. The small sample size of renal transplant patients with chronic gout reflects this. However, the results presented here are consistent with other larger studies in non-transplant populations that examined pegloticase treatment in the presence of immunomodulation (18, 19, 23). Of note, MIRROR RCT patients (pegloticase plus methotrexate vs. pegloticase monotherapy) with and without pre-treatment CKD showed stable eGFR levels (46) and possible BP decreases (40) over 52-weeks of pegloticase treatment. This study was also limited by its relatively short post-treatment follow-up period of 3 months, during which renal and QOL benefits were largely sustained. As more patients are receiving pegloticase for longer, long-term gout and renal outcomes following therapy remains an important unanswered question. Though treatment follow-up data are presented, this study was not designed to examine optimum patient management following pegloticase discontinuation. Therefore, further studies with longer post-treatment observation are needed in both transplant and non-transplant populations.

In summary, the secondary and exploratory PROTECT clinical trial endpoints offer insight on the effect of intensive urate-lowering on renal function and QOL in KT recipients. Renal function remained stable during treatment and decreases in BP during treatment were also observed. Further, pegloticase treatment was accompanied by clinically meaningful improvements in all QOL measures examined. Overall, the PROTECT clinical trial findings emphasize the high rate of SU-lowering efficacy with pegloticase among immunosuppressed KT recipients, with additional beneficial physiological effects that corresponded with the study treatment and beyond.

## Data Availability

The original contributions presented in the study are included in the article/**Supplementary Material**. Further inquiries can be directed to the corresponding author. Qualified researchers may also request data from Amgen clinical studies. Complete details are available at the following: https://wwwext.amgen.com/science/clinical-trials/clinical-data-transparency-practices/clinical-trial-data-sharing-request.
