# Corrigendum: Quality of life improvements and clinical assessments in kidney transplant recipients undergoing pegloticase treatment for uncontrolled gout: findings of the phase 4 PROTECT clinical trial

**DOI:** 10.3389/fimmu.2025.1595951

**Published:** 2025-04-03

**Authors:** Abdul Abdellatif, Lin Zhao, Katie Obermeyer, Zana Vranic, Brad A. Marder, John D. Scandling

**Affiliations:** ^1^ Department of Medicine, Division of Nephrology at CLS Health and Baylor College of Medicine, Houston, TX, United States; ^2^ Rare Disease Unit, Amgen Inc. (formerly Horizon Therapeutics), Thousand Oaks, CA, United States; ^3^ Division of Nephrology, Stanford School of Medicine, Stanford, CA, United States

**Keywords:** gout, kidney transplant, urate, quality of life, renal function, blood pressure

In the published article, the reference for “The high impact of gout on lowering patient QOL is well established, and these effects are more pronounced in KT recipients” was incorrectly written as “40”. It should be “14”.

The authors apologize for this error and state that this does not change the scientific conclusions of the article in any way. The original article has been updated.

