# Supplementary material for: Quality of life improvements and clinical assessments in kidney transplant recipients undergoing pegloticase treatment for uncontrolled gout: findings of the phase 4 PROTECT clinical trial
Source: Front Immunol. 2025 Mar 13;16:1516146. doi: 10.3389/fimmu.2025.1516146 (PMC12003961; doi:10.3389/fimmu.2025.1516146)
Supplement: Supplementary file 1 [file DataSheet1.docx]

**Supplementary Figure 1. Patient disposition in the PROTECT trial.**


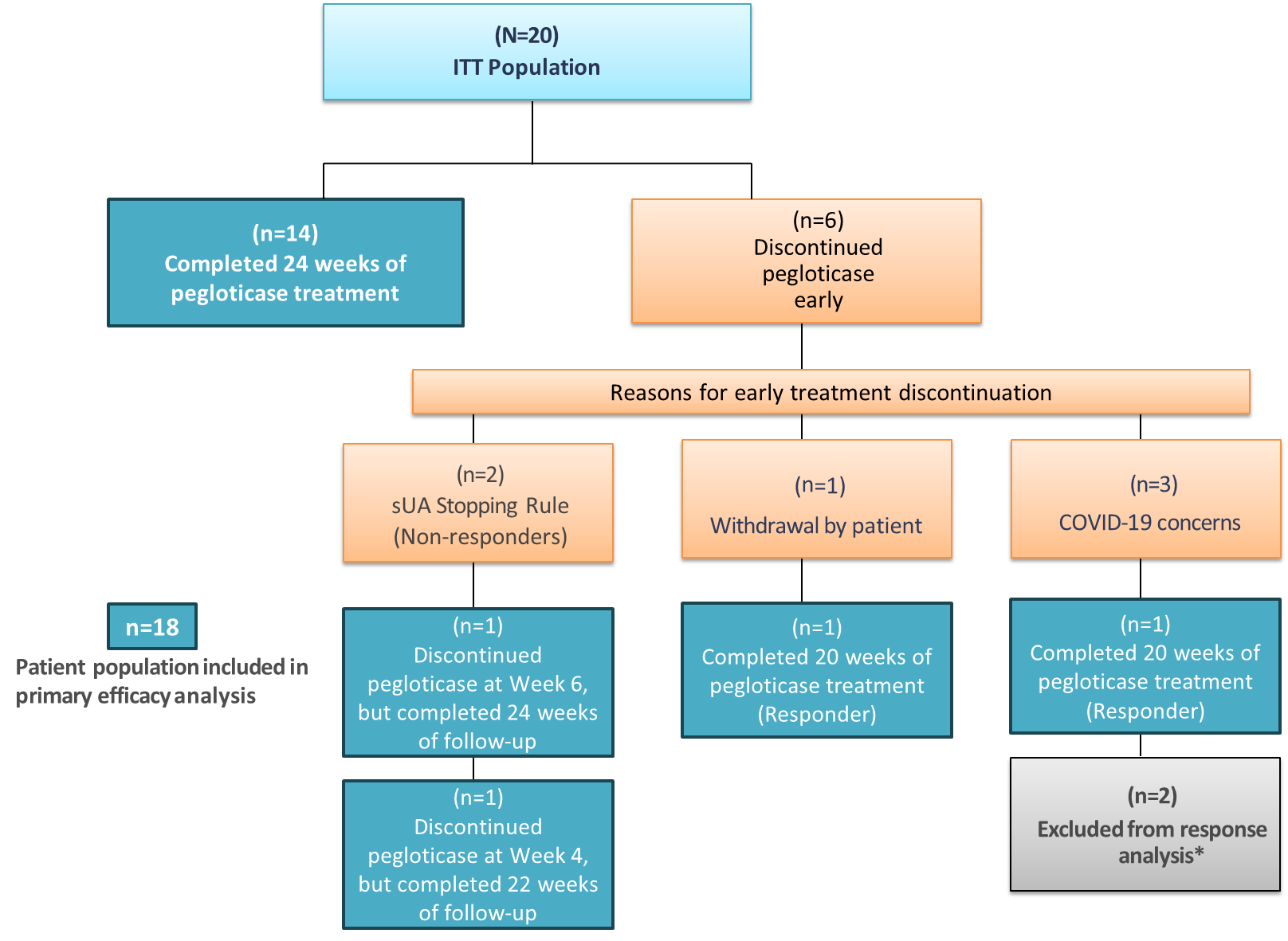


*One subject discontinued treatment after Week 2 pegloticase infusion, the other discontinued treatment after Week 6 pegloticase infusion.

The blue boxes represent patients who were included in the primary efficacy analysis.

Reference

1. Abdellatif A, Zhao L, Chamberlain J, Cherny K, Xin Y, Marder BA, et al. Pegloticase efficacy and safety in kidney transplant recipients; results of the phase IV, open-label PROTECT clinical trial. *Clin Transplant.* 2023;37(9):e14993.

**Supplementary Table 1. Prior, Concomitant, and Follow-up Medications in the PROTECT Trial ITT Population (N=20)**

| **ATC Level 4**  **Preferred Term** | **Prior** | **Concomitant with Pegloticase** | **Follow-up** |
| --- | --- | --- | --- |
| Patients with any medications | 20 (100.0) | 20 (100.0) | 20 (100.0) |
| Inhibitors, plain | 4 (20.0) | 4 (20.0) | 4 (20.0) |
| Enalapril | 1 (5.0) | 1 (5.0) | 1 (5.0) |
| Lisinopril | 3 (15.0) | 3 (15.0) | 3 (15.0) |
| Acetic acid derivatives and related substances | 1 (5.0) | 1 (5.0) | 1 (5.0) |
| Indometacin | 1 (5.0) | 1 (5.0) | 1 (5.0) |
| Adrenergics in combination with corticosteroids or other drugs (excluding anticholinergics) | 0 | 1 (5.0) | 1 (5.0) |
| Fluticasone propionate;  Salmeterol xinafoate | 0 | 1 (5.0) | 1 (5.0) |
| Aldosterone antagonists | 1 (5.0) | 1 (5.0) | 1 (5.0) |
| Spironolactone | 1 (5.0) | 1 (5.0) | 1 (5.0) |
| Angiotensin II antagonists, plain | 7 (35.0) | 7 (35.0) | 7 (35.0) |
| Losartan | 6 (30.0) | 6 (30.0) | 6 (30.0) |
| Valsartan | 1 (5.0) | 1 (5.0) | 1 (5.0) |
| Benzothiazepine derivatives | 2 (10.0) | 2 (10.0) | 2 (10.0) |
| Diltiazem | 2 (10.0) | 2 (10.0) | 2 (10.0) |
| Beta blocking agents | 1 (5.0) | 1 (5.0) | 1 (5.0) |
| Timolol maleate | 1 (5.0) | 1 (5.0) | 1 (5.0) |
| Beta blocking agents, selective | 6 (30.0) | 6 (30.0) | 5 (25.0) |
| Atenolol | 1 (5.0) | 1 (5.0) | 1 (5.0) |
| Bisoprolol | 1 (5.0) | 1 (5.0) | 0 |
| Esmolol | 0 | 0 | 1 (5.0) |
| Metoprolol | 3 (15.0) | 3 (15.0) | 3 (15.0) |
| Metoprolol tartrate | 1 (5.0) | 1 (5.0) | 1 (5.0) |
| Dihydropyridine derivatives | 7 (35.0) | 8 (40.0) | 8 (40.0) |
| Amlodipine | 5 (25.0) | 6 (30.0) | 6 (30.0) |
| Nifedipine | 2 (10.0) | 2 (10.0) | 2 (10.0) |
| Hydrazinophthalazine derivatives | 4 (20.0) | 4 (20.0) | 4 (20.0) |
| Hydralazine | 3 (15.0) | 3 (15.0) | 3 (15.0) |
| Hydralazine hydrochloride | 1 (5.0) | 1 (5.0) | 1 (5.0) |
| Sulfonamides, plain | 12 (60.0) | 12 (60.0) | 12 (60.0) |
| Bumetanide | 1 (5.0) | 2 (10.0) | 2 (10.0) |
| Furosemide | 9 (45.0) | 9 (45.0) | 9 (45.0) |
| Metolazone | 1 (5.0) | 1 (5.0) | 1 (5.0) |
| Torasemide | 2 (10.0) | 2 (10.0) | 2 (10.0) |
| Anilides | 3 (15.0) | 4 (20.0) | 3 (15.0) |
| Paracetamol | 3 (15.0) | 4 (20.0) | 3 (15.0) |
| Anti-inflammatory preparation, non-steroidal for topical use | 1 (5.0) | 0 | 0 |
| Diclofenac | 1 (5.0) | 0 | 0 |
| Propionic acid derivatives | 1 (5.0) | 1 (5.0) | 2 (10.0) |
| Ibuprofen | 0 | 0 | 1 (5.0) |
| Naproxen sodium | 1 (5.0) | 1 (5.0) | 1 (5.0) |
| Natural opium alkaloids | 1 (5.0) | 2 (10.0) | 3 (15.0) |
| Hydrocodone | 1 (5.0) | 1 (5.0) | 2 (10.0) |
| Oxycodone | 0 | 1 (5.0) | 1 (5.0) |
| Opioids in combination with non-opioid analgesics | 2 (10.0) | 2 (10.0) | 1 (5.0) |
| Oxycodone hydrochloride; paracetamol | 1 (5.0) | 1 (5.0) | 0 |
| Oxycodone; paracetamol | 1 (5.0) | 1 (5.0) | 1 (5.0) |
| Other analgesics and anti-pyrectics | 6 (30.0) | 7 (35.0) | 7 (35.0) |
| Gabapentin | 6 (30.0) | 7 (35.0) | 7 (35.0) |
| Other opioids | 1 (5.0) | 2 (10.0) | 3 (15.0) |
| Tramadol | 1 (5.0) | 2 (10.0) | 3 (15.0) |
| Corticosteroids moderately potent (Group II) | 1 (5.0) | 2 (10.0) | 2 (10.0) |
| Desonide | 1 (5.0) | 1 (5.0) | 1 (5.0) |
| Triamcinolone | 1 (5.0) | 1 (5.0) | 1 (5.0) |
| Triamcinolone acetonide | 0 | 1 (5.0) | 1 (5.0) |
| Corticosteroids, plain | 1 (5.0) | 1 (5.0) | 1 (5.0) |
| Prednisolone | 1 (5.0) | 1 (5.0) | 1 (5.0) |
| Preparations inhibiting uric acid production | 18 (90.0) | 3 (15.0) | 4 (20.0) |
| Allopurinol | 17 (85.0) | 3 (15.0) | 4 (20.0) |
| Febuxostat | 2 (10.0) | 0 | 1 (5.0) |
| Preparations with no effect on uric acid metabolism | 14 (70.0) | 13 (65.0) | 13 (65.0) |
| Colchicine | 14 (70.0) | 13 (65.0) | 13 (65.0) |

ATC, Anatomical Therapeutic Chemica; ITT, intent-to-treat.
